# Performance Evaluation of Wearable Sensor Systems: A Case Study in Moderate-Scale Deployment in Hospital Environment

**DOI:** 10.3390/s151024977

**Published:** 2015-09-25

**Authors:** Wen Sun, Yu Ge, Zhiqiang Zhang, Wai-Choong Wong

**Affiliations:** 1 Department of Electrical and Computer Engineering, National University of Singapore, Singapore 119613, Singapore; E-Mail: wong_lawrence@nus.edu.sg; 2 Institute for Infocomm Research, Agency for Science, Technology and Research, Singapore 138632, Singapore; E-Mail: geyu@i2r.a-star.edu.sg; 3 Department of Computing, Imperial College, London W120NN, UK; E-Mail: zhiqiang.snarc@gmail.com

**Keywords:** wearable sensor system, body sensor network, inter-user interference, interference mitigation

## Abstract

A wearable sensor system enables continuous and remote health monitoring and is widely considered as the next generation of healthcare technology. The performance, the packet error rate (PER) in particular, of a wearable sensor system may deteriorate due to a number of factors, particularly the interference from the other wearable sensor systems in the vicinity. We systematically evaluate the performance of the wearable sensor system in terms of PER in the presence of such interference in this paper. The factors that affect the performance of the wearable sensor system, such as density, traffic load, and transmission power in a realistic moderate-scale deployment case in hospital are all considered. Simulation results show that with 20% duty cycle, only 68.5% of data transmission can achieve the targeted reliability requirement (PER is less than 0.05) even in the off-peak period in hospital. We then suggest some interference mitigation schemes based on the performance evaluation results in the case study.

## 1. Introduction

Wearable sensor systems have been attracting intense research interest over the recent years due to their potential in practical applications such as healthcare monitoring, sports training, and interactive gaming [1,2]. A wearable sensor system can be also referred to as body sensor network (BSN), which comprises multiple sensor nodes and a coordinator worn on a human body. The physiological information of the human body collected by the sensor nodes is first delivered to the coordinator, which then forwards the information to a remote server through a network interface for further processing [3,4]. For the rest of the paper, we will denote a BSN for a wearable sensor system.

Due to the presence of the human body, BSNs have some stringent requirements for communication systems:

- **Quality of service (QoS)**: QoS measures the overall performance of a wireless network in terms of throughput, transmission delay, error rates, bandwidth, *etc.* [5,6]. In BSNs, where health and motion information are monitored in real-time, QoS requirements are strict.
- **Energy efficiency**: Sensor nodes in BSNs are typically battery-powered, and difficult to charge especially for implanted sensor nodes [7]. An energy efficient communication protocol conserves energy by reducing protocol overhead, retransmission, and collisions, and thus extends the lifetime of a BSN [8,9].
- **Heterogeneous data rate**: Heterogeneous sensor nodes are employed in BSNs with various data rate requirements (e.g., 5 kbps for Electrocardiograph (ECG) and Electroencephalography (EEG), and 1 kbps for temperature sensor, respiratory sensor, and pulse sensor [10]). A flexible communication system for BSNs should accommodate the traffic with heterogeneous data rate requirement.

The performance of BSNs may deteriorate due to a number of factors including inter-user interference, which refers to the interference caused by the simultaneous transmissions from wireless BSNs in close proximity to each other. When the sensor nodes transmit data in their respective BSNs at the same time in vicinity, interference is introduced resulting in lost or erroneous reception. As a result, throughput is reduced and energy consumption is increased in BSNs. There has been extensive research works on inter-user interference recently. Natarajan *et al*. [11] highlighted the effect of the inter-user interference on BSNs from the perspective of network architectures. They found that packet delivery rate is reduced by 35% in the presence of eight or more interfering BSNs, and thus the inter-user interference problem has to be addressed carefully. Some existing works on the design of mitigation methods for inter-user interference can be found in [12,13]. Wu *et al*. [13] proposed a method where each BSN measures the interference from other BSNs and then selects a suitable channel and transmission power. However, the computational complexity of this algorithm is high which makes it difficult to implement in realistic BSN systems due to the low available computing power in such systems. Silva *et al*. [12] presented a clear method to reduce the inter-user interference, where BSNs are reallocated channels by a fixed infrastructure when they move into the radio range of each other. This scheme reduces interference effectively if the number of congregated BSNs within the interference range is fewer than the number of available channels. However, besides the infrastructure cost, this approach leads to frequent channel switching, which incurs much overhead and is thus unsuitable for occasional and short-term interference. To develop an effective inter-user interference mitigation method for a certain scenario, it is important to have a thorough and comprehensive understanding of the prevalence and severity of such interference in the realistic BSN deployment scenario, as well as the factors affecting such interference. Towards to this goal, we have a profound study of the inter-user interference about its prevalence and significance in realistic BSN deployment scenarios in this paper.

The main contributions of this paper are as follows: First, the significance of inter-user interference is investigated from the perspective of its prevalence and resultant performance degradation in a realistic BSN deployment. Second, the effects of BSN density, traffic load, and transmission power on the inter-user interference are studied quantitatively.

The rest of this paper is organized as follows. In Section 2, we review the related works on BSNs. Section 3 depicts network model in this paper. In Section 4, we characterize the inter-user interference in terms of packet error rate (PER). In Section 5, we describe a case study in a realistic BSN deployment in hospital and show the results on performance evaluation of the wearable sensor system. Section 6 provides discussion. Section 7 draws the conclusion.

## 2. Related Works

In this section, we first give a brief overview of the communication technologies in BSNs, then review the related works on inter-user interference.

### 2.1. Communication Technologies

In BSNs, a communication technology is required to meet the stringent requirements of BSNs (QoS, energy efficiency, scalability for heterogeneous data rate) considering the specific characteristics of BSNs. The existing technologies that are applicable in BSNs include Bluetooth, ZigBee, and IEEE 802.15.6. As Bluetooth does not support various traffic priorities in a device such as on-demand traffic, normal traffic, and emergency traffic in healthcare applications [14,15,16], we consider IEEE 802.15.4 and IEEE 802.15.6 in this paper [14,15,16].

IEEE 802.15.4 is currently the most widely used radio standard in BSNs for its very low power consumption and cost [17,18,19,20]. ZigBee is based on IEEE 802.15.4. Compared with other short range communication technologies (e.g., Bluetooth, Wi-Fi), ZigBee has the least protocol complexity and wake-from-sleep time. Moreover, it provides several power options depending on the specific applications (from -25 dBm to 0 dBm), and it supports mesh network configurations. The only drawback is that ZigBee supports a data rate of up to 250 kbps, which may be insufficient for some BSN applications.

The IEEE 802.15.6 working group has been formed to develop a standard for short-range, wireless communication for body area networks [21,22]. It supports QoS, low power, and data rates up to 10 Mbps. This standard considers effects on antennas in the presence of a human body (varying with male, female, thin, fat, *etc*.), radiation pattern shaping to minimize specific absorption of the human body, and changes in characteristics due to the BSN user motions. It defines a MAC layer that supports several physical layers, such as 2.4 GHz ISM Narrowband, ultra-wideband (UWB), and human body communications layers. Compared with IEEE 802.15.4, IEEE 802.15.6 requires a shorter communication range, and larger data rate, in order to safe more power cost, and enables support various applications. From the perspective of MAC, 802.15.4 and 802.15.6 are similar. In this paper, we will consider IEEE 802.15.4 and IEEE 802.15.6 for possible MAC in a BSN.

### 2.2. Interference Mitigation

The existing interference mitigation schemes for BSNs mainly fall into several categories: channel hopping, power control, and beacon shifting.

In IEEE 802.15.6, one of the mechanisms for mitigating inter-user interference in BSNs is channel hopping [21]. The coordinator of a BSN changes its operating channel in the operating frequency band periodically by including the current channel hopping state and next channel hop fields in its beacons. A BSN should choose a channel hopping sequence different from that of its neighboring BSNs. The drawback of channel hopping is that collisions cannot be effectively alleviated within a limited hopping bandwidth regardless of the delay and the energy consumption. Sergio *et al*. [23] proposed a channel hopping approach, where each BSN is assigned a different frequency channel at the network initialization phase. This approach allows monitoring as many patients given available channels, but radio channels are hard to be reused in a dynamic way. To increase the number of monitored BSNs and enable them to move freely, an alternative approach is to allocate channels dynamically in small-scale deployments of BSNs.

Power control is another approach to reduce the interference in multi-user environments [13,24,25,26,27]. Kim *et al*. [25] proposed a decentralized power control algorithm based on the received signal interference. The transmit power is so determined that the transmitter can sustain a high data rate, while keeping the adverse interference effect on the other neighboring concurrent transmissions minimal. Wu *et al*. [13] proposed a power control approach for interference mitigation, where each BSN measures the interference from other BSNs and then selects a suitable channel and transmission power by utilizing non-cooperative game theory. A major drawback of this method is the long iteration period (more than 20 iterations) to reach the optimal point. As such, the utilized channels and transmission powers may be changed frequently during the long computing period which makes the system unstable. Power control improves spatial utilization of channels, but it may compromise transmission performance with a reduced transmission power [27]. Because of the simple structure of a BSN and the concern for energy conservation, power control is infeasible for BSNs.

In healthcare applications, the channel utilization of a BSN is usually low for energy conservation [8,9]. In IEEE 802.15.6 [28], the coordinator of a BSN may transmit its beacons at different time offsets relative to the start of the beacon periods by including a beacon shifting sequence field in its beacons [29,30]. However, it is challenging to keep a beacon shifting sequence mechanism in the mobile BSN scenario. Kim *et al*. [31] proposed a distributed flexible beacon schedule scheme to reduce the interference. By employing carrier sensing before each beacon transmission, collisions can be avoided if other BSNs attempt to access the channel at the same time. This scheme consumes additional energy in the channel access as multiple carrier sensing iterations are possibly conducted before each beacon transmission.

In order to develop an effective interference mitigation scheme and interference analysis, it is imperative to have a thorough understanding of the inter-user interference. In this paper, we address the significance of the inter-user interference in a realistic BSN deployment scenario and then provide suggestions on the interference mitigation schemes.

## 3. Network Model

**Figure 1:**
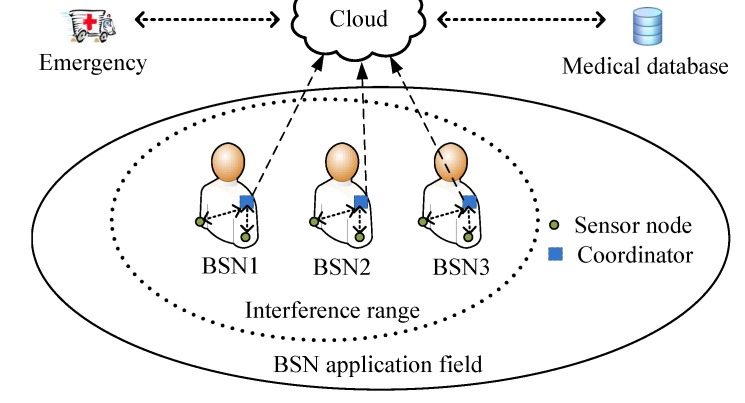
The common architecture of body sensor networks (BSNs).

Figure 1 illustrates the common architecture of BSNs. A BSN comprises multiple sensor nodes and a coordinator worn on a human body. Sensor nodes measure the physiological information of the BSN user (e.g., heart rate, blood pressure, body and skin temperature, oxygen saturation, respiration rate). The sensory data is delivered to the coordinator on the body (e.g., a personal digital assistant, a smart phone, or a micro-controller board), which then in turn displays the corresponding information on a user interface or transmits the aggregated vital signs to a remote server through a network interface for further processing [32,33]. As in typical BSN application areas, e.g., inpatient department or emergency department in a hospital, wireless local area network (WLAN) access points (APs) are commonly deployed for public Internet services, a BSN always connects to an applicable WLAN AP (as the network interface) when multiple interfaces, e.g., cellular network and WLAN, may be available.

The signal attenuates as it propagates over space in BSNs. The attenuation may either be due to propagation losses caused by the natural expansion of the radio wave in the environment, referred to as path-loss, or multi-path propagation, referred to as multi-path induced fading, or due to shadowing from obstacles affecting the wave propagation, sometimes referred to as shadow fading [34,35]. In this study, we consider the path-loss and shadow fading for simplicity. The path-loss function is given by $l\left(d\right)=G\cdot d^{-\alpha }(d>d_{0})$, where *d* is the distance between the transmitter and receiver, *G* is a constant accounting for system loss, α is the path-loss exponent with α>2, and $d_{0}$ is the reference distance. Denote the shadow fading factor as *γ*. At a given time *t*, the received signal strength is expressed as

(1) $$\Omega \left(d\right)=\Omega_{0}\cdot \gamma \cdot l\left(d\right)$$

where $\Omega_{0}$ is the transmission power.

In BSNs, there are two different channel models, *i.e*., the wireless channel between the sensor nodes and the coordinator in a BSN (referred to as on-body channel model) and that between two BSNs (referred to inter-body channel model). Due to the blockage and disruption of the radio signal by the human body, the on-body channel model experiences more severe attenuation (with a path-loss exponent $\alpha_{O}$) than the inter-body channel model (with a lower path-loss exponent $\alpha_{I}$ and $2<\alpha_{I}<\alpha_{O}$) [36,37].

The transmission status of a BSN could be transmitting or not transmitting. The transmission status is 1 when there is any node, either sensor node or coordinator node, transmitting. Then transmission status is 0 when none of the node transmits, it is inactive period in a superframe.

## 4. Interference Characterization

The inter-user interference of wearable electronics is caused by a number of factors, such as distance between networks, human effects, and environmental effects. In the paper, we model the effects of human effects by observing the locations of BSN users and interaction between them in the waiting area of a hospital. Moreover, we model the channel on a human body by introducing an on-body communication channel model and an inter-body communication channel model, where the channel characteristics are set through experimental results. There are several metrics to depict the performance of a BSN, such as bit error rate, packet error rate, and throughput.

The following terms are utilized to describe our model:

- ***Signal to interference and noise ratio (SINR)*** is the ratio of the desired signal power received from transmitter to the total power from interferers and receiver noise.
- ***Packet error rate*** is the number of incorrectly received data packets divided by the total number of received packets.
- ***Spatial throughput*** measures the number of BSNs that transmit simultaneously and successfully within a unit area. It measures the overall performance of BSNs.
- ***Spatial average rate*** is a measure of the quantity of users or services that can be simultaneously supported by a limited radio frequency bandwidth in a defined geographic area.

The notations and symbols involved in this paper are presented in Table 1.

**Table 1:** The notations of the selected terms.

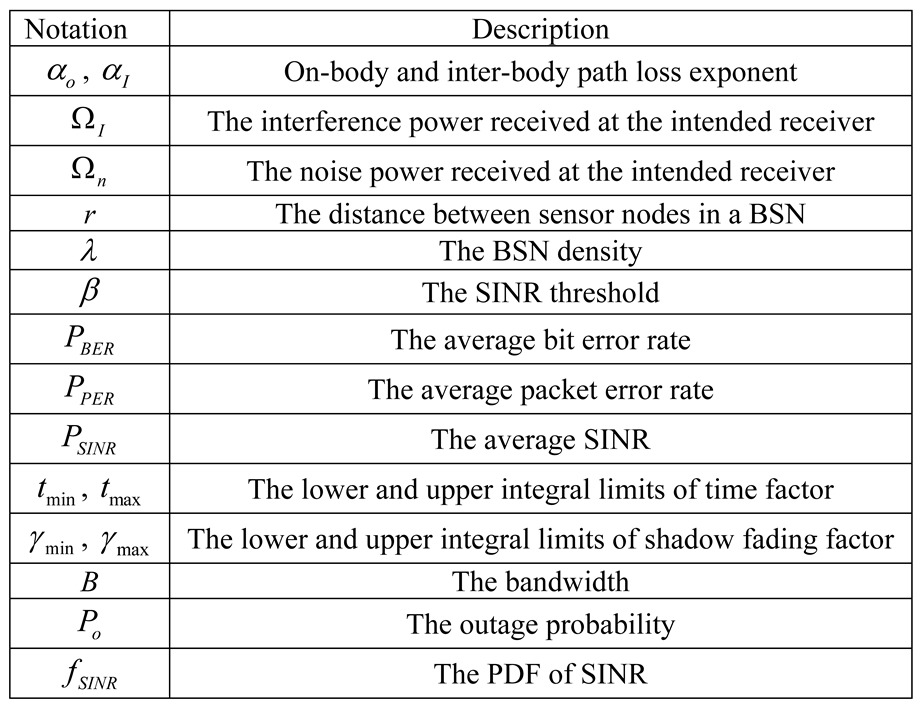

### 4.1. Signal to Interference and Noise Ratio (SINR)

Data transmissions of a BSN incur interference to its neighboring BSNs, referred to as inter-user interference. Packet error rate (PER) is utilized to characterize the level of service degradation of BSNs due to inter-user interference. PER is defined as the ratio of the number of incorrectly received data packets to the total number of transmitted packets.

PER can be derived from the signal-to-interference noise ratio (SINR) of a BSN. Note that not all the neighboring BSNs transmit at time *t*. We denote ${𝟙}_{j}\left(t\right)$ as the transmission status of BSN *j* at *t*. ${𝟙}_{j}\left(t\right)=1$ when BSN *j* transmits at time *t*, and ${𝟙}_{j}\left(t\right)=0$ when BSN *j* does not transmit at *t*. It is noted that the probability of ${𝟙}_{j}\left(t\right)=1$ also represents the duty cycle of BSN *j*.

The SINR of BSN *i* at time *t* is expressed as,

(2) $${P_{SINR}}_{i}\left(t\right)=\frac{\Omega (r_{i}\left(t\right))}{\sum_{j\neq i}{𝟙}_{j}\left(t\right)\cdot \Omega \left(u_{i,j}\left(t\right)\right)+\Omega_{n}}$$

where $r_{i}\left(t\right)$ is the distance between the transmitting sensor and the coordinator for the BSN *i* at time *t*, $u_{i,j}\left(t\right)$ is the distance between the transmitting node of BSN *j* and the coordinator of BSN *i* at *t*, and $\Omega_{n}$ is the background noise power [38].

### 4.2. Packet Error Rate

*Packet error ratio (PER)* is the number of incorrectly received data packets divided by the total number of received packets. A packet is declared incorrect if at least one bit is erroneous. When offset quadrature phase shift keying (OQPSK) (Adopted by the PHY in IEEE 802.15.4 standard at 2.4 GHz [39].) is utilized as the modulation scheme, the average bit error rate (BER) and PER for BSN *i* can be expressed as,

(3) $$P_{BER}=\frac{1}{2}erfc\left(\sqrt{2E_{b}/N_{0}}\right)=\int_{t_{min}}^{t_{max}}\int_{\gamma_{min}}^{\gamma_{max}}\frac{1}{2}erfc\left(\sqrt{{P_{SINR}}_{i}\left(t\right)}\right)\cdot P\left(\gamma \right)d\gamma dt$$

(4) $$P_{PER}=\sum_{a=1}^{m+k-m/2}C_{m+k}^{a+m/2}\cdot {P_{BER}}^{\left(a+m/2\right)}\cdot {\left(1-P_{PER}\right)}^{\left(m+k-(a+m/2)\right)}$$

where $E_{b}/N_{0}$ is SINR per bit, $erfc\left(\cdot \right)$ is the complementary error function [40], *m* is the number of information bit, *k* is the number of additional coding bit, and $C_{m+k}^{a+m/2}$ is the set of all (a+m/2) combinations out of a set (m+k), $t_{min}$ and $t_{max}$ are the lower and upper integral limits of time factor, $\gamma_{min}$ and $\gamma_{max}$ are the lower and upper integral limits of shadow fading factor [41]. Equation (3) and Equation (4) can be applied to other modulation schemes as well with little modification [42].

As can be seen from Equation (4), multiple factors affect the PER performance, including neighboring BSN number, traffic load, and transmission power. The effects of those factors are investigated in the case study.

### 4.3. Outage Probability

In the presence of inter-user interference, outage occurs when the SINR of a BSN is below an acceptable threshold *β*, *i.e*.,

(5) $$P_{o}=\mathrm{Pr}\left\{{P_{SINR}}_{i}\left(t\right)<\beta \right\}$$

We assume the noise is white noise, *i.e*., being constant over the whole frequency band.

Denote the CDF of SINR regarding β as $F_{SINR}\left(\beta \right)$. We have the outage probability of a BSN regarding the SINR threshold *β* is

(6) $$\begin{array}{cc} P_{o} & =1-F_{SINR}\left(\beta \right)=1-\underset{x\geq 0,y\geq 0,x/y\leq \beta }{\;\int \int \;}f_{s}\left(x\right)f_{I}\left(y\right)dxdy\\ & =1-\int_{0}^{\infty }F_{s}\left(\beta y\right)f_{I}\left(y\right)dy \end{array}$$

where $f_{s}\left(x\right)$ is the PDF of the desired signal *x*, $f_{I}\left(y\right)$ is the PDF of the interference signal *y*, and $F_{s}\left(x\right)$ is the cumulative distribution function (CDF) of the desired signal. This is the ratio of the desired signal to the interference is less than β, *i.e*., x/y≤β, when the noise is negligible, *i.e*.,

$$\underset{x\geq 0,y\geq 0,x/y\leq \beta }{\;\int \int \;}f_{s}\left(x\right)f_{I}\left(y\right)dxdy=\int_{0}^{\infty }\int_{0}^{\beta y}f_{s}\left(x\right)f_{I}\left(y\right)dxdy$$

### 4.4. System Performance

#### 4.4.1. Spatial Average Rate

*Spatial average rate* is a measure of the quantity of users or services that can be simultaneously supported by a limited radio frequency bandwidth in a defined geographic area. It is defined as $\bar{R}=E\left[\ln\left(1+SINR\right)\right]$, where the expectation is with respect to the random fading channels and the random positions of the transmitter nodes.

Assume that the PDF of SINR is $f_{SINR}\left(x\right)$. The spatial average rate can be expressed as

(7) $$\begin{array}{cc} & \int_{x=0}^{\infty }\ln\left(1+x\right)f_{SINR}\left(x\right)dx\\ = & \int_{z=0}^{\infty }\frac{1}{1+z}dz\int_{x=z}^{\infty }f_{SINR}\left(x\right)dx\\ = & \int_{z=0}^{\infty }\frac{1-P_{o}\left(z\right)}{1+z}dz \end{array}$$

Equation (7) is achieved because

$$F_{SINR}\left(z\right)=\int_{x=0}^{z}f_{SINR}\left(x\right)dx=P_{o}$$

according to the definition of outage probability.

#### 4.4.2. Spatial Throughput

A specific metric in stochastic geometry [43] is *spatial throughput* which characterizes the density of the nodes which successfully transmit at a given time (*i.e*., BSNs in the context of this paper) within a unit area. Spatial throughput considers the successful transmission probability of a BSN as well as the spatial reuse. For example, according to [43], in the case of Aloha MAC network with half-duplex transceivers, the spatial throughput is expressed as $p\left(1-p\right)\left(1-P_{o}\right)$ [43], where *p* is spatial transmission probability, $\left(1-p\right)$ is the probability that the intended receiver is listening (not transmitting) when the transmitter transmits, and $\left(1-P_{o}\right)$ is the successful transmission probability. In a BSN where the receiver is in listening state when the transmitter is transmitting, the term $\left(1-p\right)$ is negligible. Moreover, we utilize the intensity of interfering BSNs, e.g., *λ*, instead of the spatial probability *p* in order to consider the ratio of contention-based traffic and contention-free traffic in IEEE 802.15.6 BSNs.

The spatial throughput is given by

(11) $$S=\lambda \left(1-P_{o}\right)$$

Both spatial throughput and spatial average rate measure the overall performance of BSNs. From Equation (7) and Equation (8), both metrics consider the outage probability $P_{o}$, while spatial average rate does not consider the intensity of nodes. The area spectral efficiency is shown to follow the similar trend with the spatial throughput.

## 5. Case Study

In order to study the severity and prevalence of inter-user interference, a case study has been performed in the waiting area of Emergency Department of the National University Hospital (NUH) of Singapore.

### 5.1. Deployment Scenario

**Figure 2:**
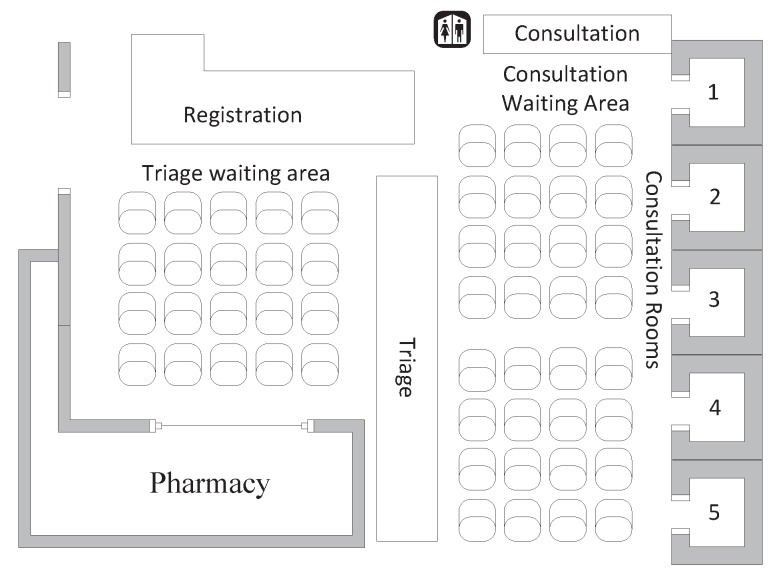
Floor map of waiting area in National University Hospital (NUH) Emergency Department.

Figure 2 shows the floormap of NUH Emergency Department waiting area. The standard operational process of NUH emergency is as follows: (1) the patient first registers at the registration counter; (2) the patient then waits for triage at the triage waiting area; (3) after the triage, the patient waits at the consultation waiting area to get preliminary consultations at the consultation counter and later treated in one of the consultation rooms by doctors. When BSNs are deployed in the Emergency Department of a hospital, BSNs will be operational during processes (2) and (3).

From the statistics of the NUH Emergency Department, the total waiting time a patient spends at the waiting area of Emergency Department of NUH is one to two hours on average for different visiting periods, *i.e*., peak or off-peak time. Because a patient’s status may deteriorate during the waiting time, it is necessary to monitor critical physiological data of an unattended patient through a BSN in the waiting areas. Once any emergency situation is detected, the patient should be given priority to be treated [44,45,46].

### 5.2. Experimental Settings

We observe the distribution of the patients in the NUH Emergency Department waiting area for three days. The observation period is 15 h per day from 08:00 to 23:00. It is found that the observation periods of a day can be divided into three categories:

- Peak time (8:00 to 10:30): 58 BSNs are in the waiting area on average, thus the average area per BSN is 2 square meters per person;
- Moderate time (10:30 to 21:00): 40 BSNs are in the waiting area on average, thus the average area per BSN is 3 square meters per person;
- Off-peak time (21:00 to 23:00): 16 BSNs are in the waiting area on average, thus the average area per BSN is 6 square meters per person.

In the analysis, we consider all BSNs transmitting at the same power $\Omega_{s}\in [-25$dBm,0 dBm]. The signal transmitted in the same BSN attenuates according to the on-body path loss model [36,47], while the interference signal reaches the BSN of interest according to the inter-body path loss model [37], as listed in Table 2. For simplicity, the duty cycles of all BSNs are set the same as well. In addition, we assume all the BSNs operate on the same channel. The performance of multiple available channel scenario can be easily obtained from that of the single channel scenario with the same number of BSNs per channel. In this paper, we are concerned about the performance of BSNs instead of system performance, thus consider PER of BSNs in the simulation results. Moreover, BSN users may tend to congregate sometime in some specific areas when emergency event happens. For most of the time, the BSN users tend to be seated evenly in the waiting area of the emergency department of the hospital. In this paper, we study the average interference effects for the common cases.

**Table 2:** The parameter settings of the simulation in case study.

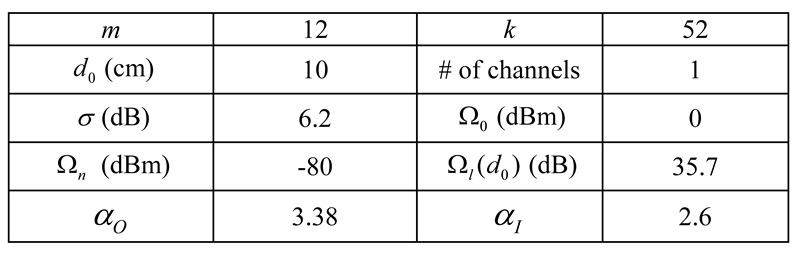

### 5.3. Simulation Results

This section investigates the effects of BSN density, traffic load, and transmission power on the BSN performance. PER of BSNs are calculated using Matlab 7.0 [48] according to Equation (4).

#### 5.3.1. Effect of BSN Density

**Figure 3:**
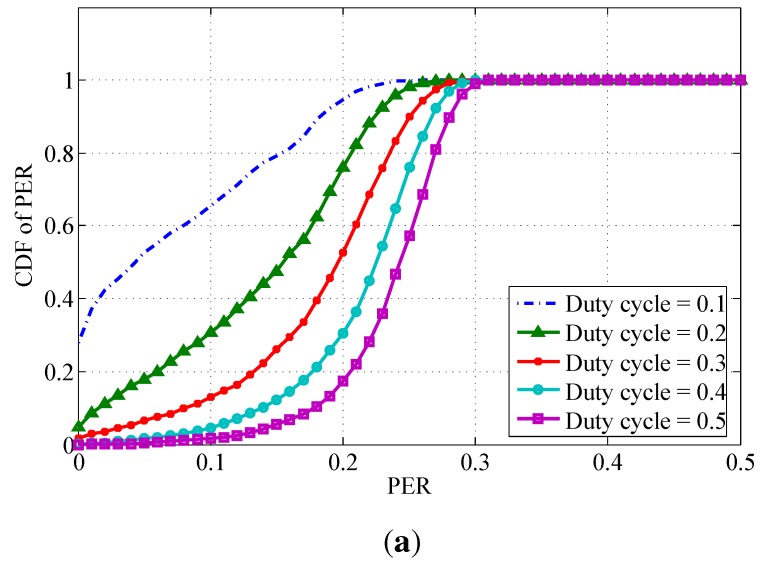

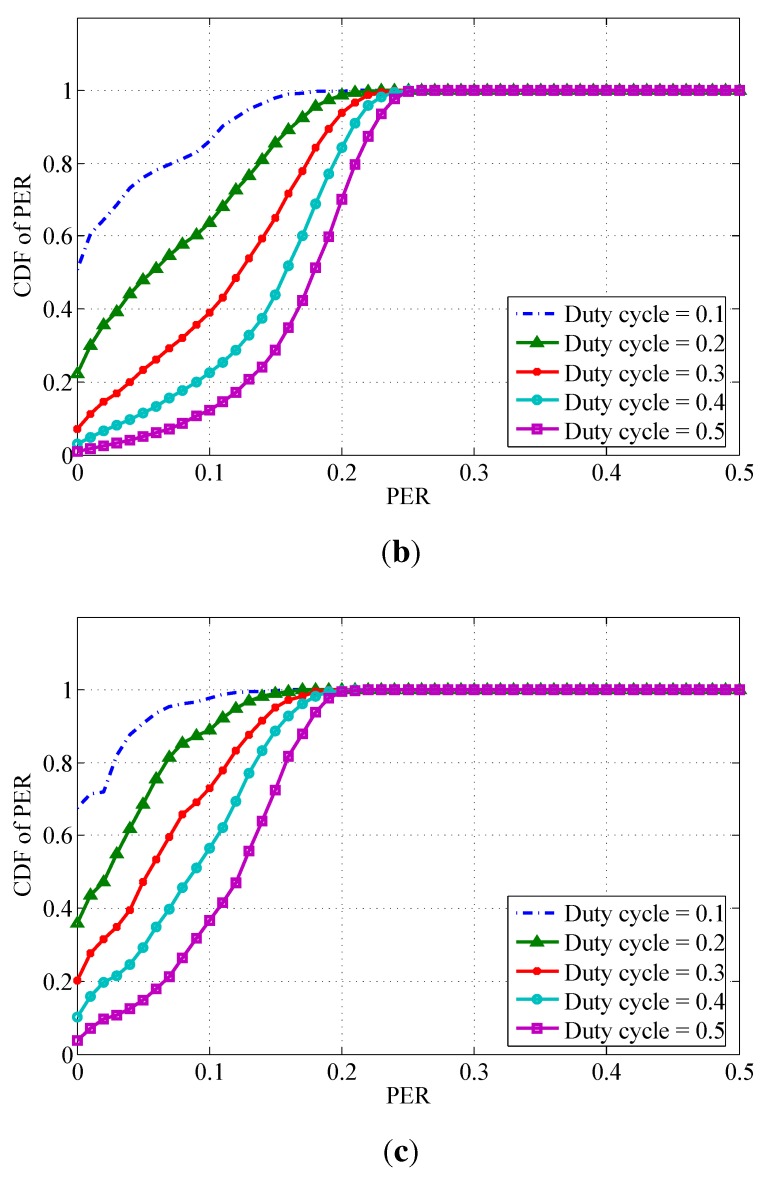
The cumulative distribution function (CDF) of packet error rate (PER) for three scenarios. (**a**) Peak time scenario; (**b**) Moderate time scenario; (**c**) Off-peak time scenario.

Figure 3 shows the CDF of PER in the peak time scenario, moderate time scenario, and off-peak time scenario respectively. Each curve shows the CDF of PER with a specified duty cycle. The PER increases with the duty cycle. This is reasonable because when the duty cycle gets higher more BSNs transmit simultaneously resulting in higher PER. When comparing the three graphs in Figure 3, the PER decreases as the BSN density decreases from 2 square meters per person in peak period (see Figure 3a) to 6 square meters per person in off-peak period (see Figure 3c). This is caused by less severe interference due to lower density BSNs.

**Table 3:** Reliability level (%) with PER lower than 0.05.

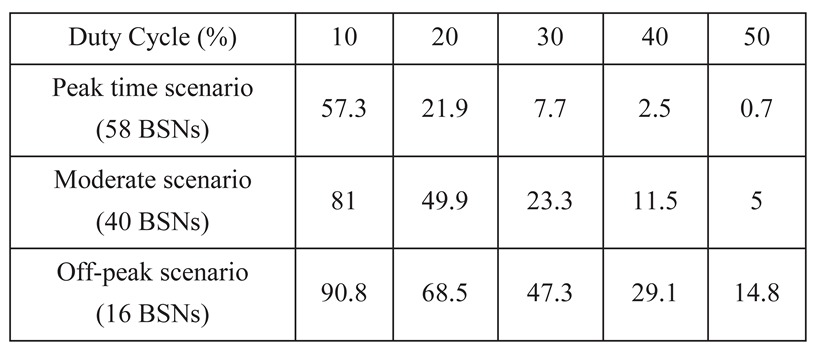

From Figure 3, we obtain the reliability level of data transmission in BSNs. The reliability level is defined as the ratio of the number of successfully received data packets (with PER less than a certain target PER requirement $PER_{required}$) to the number of transmitted data packets. The reliability level can be calculated as $\mathrm{Pr}(PER<PER_{required})$. Assuming that reliability requires PER being less than 0.05 ($PER_{required}=0.05$), the reliability level is listed in Table 3. For instance, in the case of *Duty cycle = 0.2*, under the scenario of peak time, the reliability level is only 21.9%. At the off-peak time, the reliability level is 68.5%.

#### 5.3.2. Effect of Transmission Power

**Figure 4:**
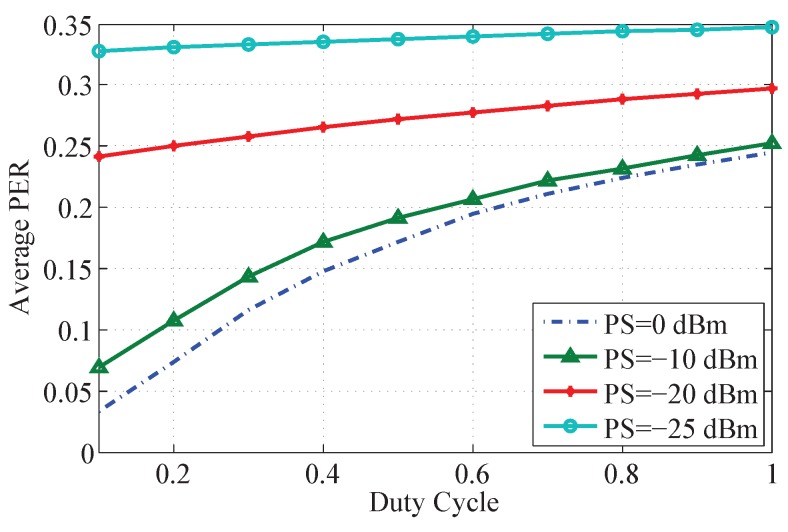
Average PER for different transmission power, where PS represents the transmission power.

Figure 4 shows the PER of BSNs under different transmission power settings. The PER is obtained by averaging over all the BSNs in the moderate time period at the respective transmission power. As can be seen in Figure 4, the average PER increases when the transmission power gets lower. In particular, when the transmission power is −25 dBm, the average PER is 54% higher than that of 0 dBm. It is also found that the PER difference caused by duty cycles becomes less obvious when the transmission power gets lower. This is because when the transmission power is low, the main reason for erroneous or loss of packets is the radio signal blockage by the human body rather than inter-user interference. In comparison, when the transmission power is sufficiently high, the packets loss is mainly due to inter-user interference.

#### 5.3.3. Effect of on-Body Path Loss Model

**Figure 5:**
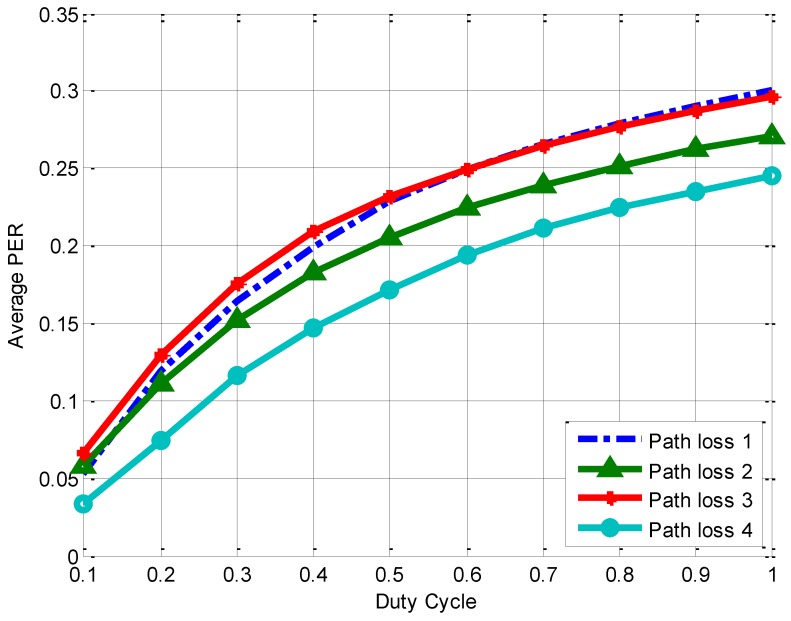
Average PER for different on-body path loss model.

Given the heavy attenuation of the human body to communication at 2.4 GHz, node placement is expected to play a significant role in the PER performance. Moreover, on-body path loss model differentiates depending on the body size of BSN wearer. To investigate the effect of on-body path loss model we compare the inter-user interference under three different node placement. The path loss model parameters are listed in Table 4. Figure 5 shows the average PER over different node placement. Thus on-body path loss model influences the inter-user interference obviously. In order to increase probability of successful packets, node placement on the same side of body is preferred.

**Table 4:** The parameter settings of the path loss model in case study.

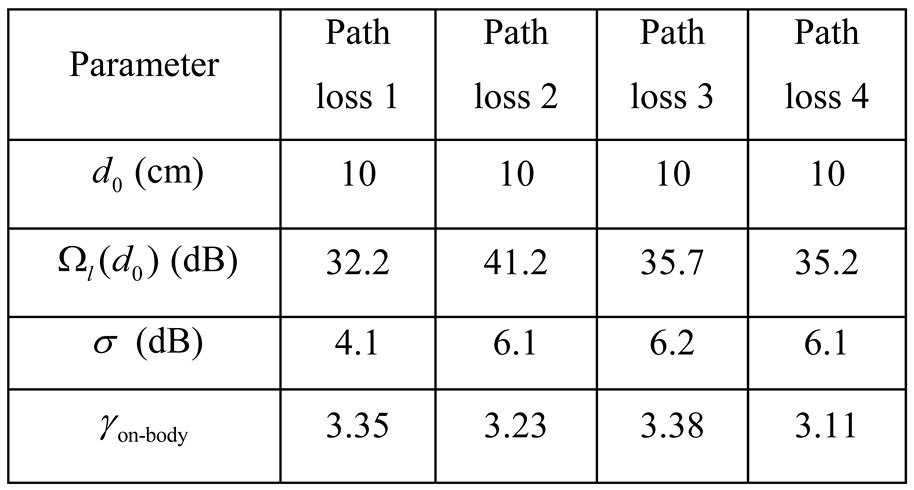

## 6. Discussion

This interference investigation provides insights and guidelines for BSN deployment configurations, such as the maximum traffic load of a BSN and the maximum BSN density, to meet the reliable communication requirements. For instance, in the moderate scenario, given the reliability requirement (PER is less than 0.05) and the reliability level (at least 90% of the data packets are transmitted reliably), the duty cycle of a BSN should be less than 10% (obtained from Table 3). Conversely, given that the duty cycle of a BSN is 10% and the reliability level is 90%, the maximum number of BSNs that can be accommodated on one channel is around 16 in the off-peak scenario (also obtained from Table 3). In addition, given the BSN density and transmission power, there is a maximum traffic load (duty cycle) for each BSN. For example, from Figure 4, given the reliability requirement (PER is less than 0.1), the maximum duty cycle of a BSN is around 30% with a transmission power of 0 dBm, while the maximum duty cycle of a BSN is around 20% with a transmission power of −10 dBm. Also from Figure 4, we know that when the transmission power is low, the main reason for erroneous or lost packet reception is the radio signal blockage by the human body rather than inter-user interference. Thus a minimum transmission power has to be ensured to avoid packet loss by blockage of human body. This is under the assumption that intra-BSN communication in a BSN is coordinated by the coordinator of the BSN following a centralized MAC. There are cases that BSNs employ a distributed MAC without a coordinator. As long as there is only one sensor node transmitting in a BSN at a time, the analysis results in the paper work. For the distributed intra-BSN MAC cases, we would suggest CSMA/CA for the interference mitigation scheme. The cases of multi-channel scenarios actually could be obtained easily from the single channel case by just considering the same number of networks on a single channel. Inter-user interference could be determined by a number of factors including distance between BSNs, human effects, and environmental effects. It is difficult to evaluate the performance of BSNs without specific application settings. According to our experimental experiences, when the experimental environment changes, the performance of a BSN could be 23% worse than the studied case.

In addition, the investigation provides implications on mitigation schemes for inter-user interference in BSNs when the multitude of operating scenarios is predetermined. For instance, we may reduce the probability of overlapping transmission to improve the SINR. To achieve this goal, transmissions of neighboring BSNs can be rescheduled to avoid overlapping transmissions when the BSN density is low. Moreover, when the BSN density is high, exceeding the maximum number of BSNs that can be accommodated, a channel switching scheme can be incorporated to reduce the congestion level of the current channel. In addition, each BSN is able to select its transmission power adaptively according to its surrounding environment and performance requirement. The BSN with higher priority use higher transmission power to ensure its reliable transmission. In the future work, we proposed a lightweight and distributed inter-user interference mitigation scheme, that can be easily integrated with the IEEE 802.15.4 protocol stack. The proposed scheme takes into consideration the generic property of low channel utilization in BSNs and enables affected BSNs to adaptively reschedule their transmission time or switch channels.

## 7. Conclusions

In this paper, we have investigated the significance of the inter-user interference in a realistic moderate-scale wearable sensor system scenario in hospital. Simulation results showed that with the target packet error rate (PER) requirement (e.g., 0.05), the reliability level of BSN transmission is only 21.9% at the peak time. Even at the off-peak time, the reliability level is only 68.5%. Thus inter-user interference exists widely and severely in the BSN deployment scenario. Based on the investigation, the BSN deployment configurations such as maximum BSN number and the maximum traffic load are implicated. In addition, inter-user interference mitigation schemes are suggested based on the specified scenario.
